# Alleviating effects of dietary formic acid polymer supplementation on lipopolysaccharide-induced inflammatory injury in gut-liver axis of broilers

**DOI:** 10.1016/j.psj.2024.104698

**Published:** 2024-12-19

**Authors:** Guohui Zhou, Yuemeng Fu, Jiali Chen, Weiren Yang, Ning Jiao, Shuzhen Jiang, Xuejun Yuan, Yang Li

**Affiliations:** a Key Laboratory of Efficient Utilization of Non-Grain Feed Resources, College of Animal Science and Technology, Ministry of Agriculture and Rural Affairs, Shandong Agricultural University, Panhe Street 7, Tai'an 271017, China; b College of Life Sciences, Shandong Agricultural University, Daizong Street 61, Tai'an 271017, China

**Keywords:** Broiler, Formic acid, Inflammation, Gut-liver axis

## Abstract

A close interplay exists between the gut and liver, known as the "gut-liver axis", which plays a vital role in health and disease. This research aimed to explore the effects of dietary formic acid polymer (FAP) addition on inflammatory injury in gut-liver axis of broilers due to lipopolysaccharide (LPS) challenge. Four hundred and fifty 1-day-old male Arbor Acres broilers were assigned to three treatment groups: (1) control (non-challenged, basal diet); (2) LPS (LPS-challenged, basal diet); (3) LPS+FAP (LPS-challenged, basal diet with 1,000 mg/kg FAP). The trial lasted 21 days. On experimental days 17, 19, and 21, the LPS and LPS+FAP groups were intraperitoneally administered LPS (1 mg/kg BW), and the control group received an equal volume of physiological saline via intraperitoneal injection. Results showed that LPS injection induced inflammatory response, resulted in liver damage, and destroyed intestinal morphology and mucosal barrier. However, dietary FAP supplementation alleviated LPS-induced adverse effects on liver and small intestine by decreasing inflammatory response and suppressing cell death. In conclusion, supplementation of 1000 mg/kg FAP mitigated LPS-induced inflammatory injury in gut-liver axis in broilers.

## Introduction

The liver is a central metabolic organ and a crucial mediator of immunological functions, playing important roles in nutrients metabolism, endotoxin clearance, and defense against gut-derived antigens. However, modern broiler farming, characterized by high-density housing, rapid growth rates, and the use of antibiotics and other additives, significantly increases the risk of liver dysfunction, excessive fat accumulation, and liver injury (Caekebeke et al., 2020). Hepatic inflammatory injury caused by pathogen invasion is a common clinical disease, which could lead to the reduction in growth performance and even death. Positioned between the absorptive surfaces of the gastrointestinal tract, the liver receives approximately 75 % of its blood supply from the small and large intestines via the portal circulation. This positioning underscores the liver's role as the primary organ to encounter enterally absorbed nutrients and microbial metabolites from the intestine (Hsu and Schnabl, 2023). Furthermore, the integrity of the intestinal barrier is crucial not only for nutrient absorption but also for confining trillions of microbes within the lumen, thereby preventing their systemic dissemination. Once the intestinal barrier is compromised, endotoxins and pathogens will enter the body, causing damage to the liver ultimately (Hsu and Schnabl, 2023). It has been proven that gut-liver axis is involved in the repairment of liver disease in broilers (Tao et al., 2023). Therefore, improving the intestinal barrier has the potential to relieve inflammatory damage to the liver of broilers in modern intensive farming.

The application of feed acidifiers has generally been shown to improve broiler growth performance and gut morphology (Luise et al., 2020). Formic acid (**FA**) is usually used as a feed hygiene enhancer to improve feed hygiene, but FA is highly corrosive and irritating, which limits its use in poultry production. Therefore, the derivatives of FA, such as formate and potassium diformate, gradually emerged, and showed a better potential serving as alternatives to antibiotic growth promoters than FA (Luise et al., 2020). Formic acid polymer (**FAP**) is formed from two molecules of FA through polymerization process. Recent study showed that 1,000 mg/kg FAP could be used as a substitute for antibiotic in diets to enhance growth performance and liver health in Arbor Acres (**AA**) broilers (Wang et al., 2022). However, few data are currently available regarding the alleviating influences of FAP on the inflammatory injury in gut-liver axis of broilers.

Lipopolysaccharide (**LPS**) is a potent immune system stimulator found in the structural component of the outer membrane of Gram-negative bacteria. Previous studies indicated that LPS could reduce the performance, disrupt intestinal flora, and cause intestinal and liver inflammatory injury (Han et al., 2020; Liu et al., 2024). Therefore, the LPS was employed in this study to construct the gut-liver inflammation model, and to explore the alleviating effects of dietary FAP addition on LPS-induced inflammatory injury in gut-liver axis of broilers, verifying whether intestinal barrier was involved in the mitigating effects of FAP on liver inflammatory injury.

## Materials and methods

### Ethical statement

The current study was carried out at the Research Farm of Shandong Agricultural University. All the animals and procedures were approved by the Care and Use Committee of Shandong Agricultural University (ethical code SDAUA-2023-173).

### Animals and treatments

Four hundred and fifty 1-day-old male AA broilers (average BW 48.47 ± 0.46 g) were randomly divided into three treatment groups with 6 replicates of 25 broilers for a 21-day trial. The groups were: (1) control (non-challenged, basal diet); (2) LPS (LPS-challenged, basal diet); (3) LPS+FAP (LPS-challenged, basal diet with 1,000 mg/kg FAP). The basal diet was formulated in compliance with the NRC (1994), as referenced by our previous study (Liu et al., 2024). Broilers were kept in three-level cages (120 cm × 70 cm × 40 cm), and had unlimited access to water and feed. On days 17, 19, and 21 of the experiment, broilers in the LPS and LPS+FAP groups received intraperitoneal injections of 1 mg/kg BW *E. coli* O55:B5 LPS (Chen et al., 2018), while those in the CON group were injected with an equal amount of saline solution (0.9 % sodium chloride).

### Sampling

On day 21 of the trial, 3 h after LPS challenge, six broilers from each group (one per replicate) were selected for blood sampling via the wing veins to obtain serum. Subsequently, the broilers being euthanized, liver specimens (1 × 1 × 0.5 cm³) and 2-cm segments from the midsection of the small intestine were harvested, rinsed with 0.9 % saline solution, and fixed in 4 % paraformaldehyde for 24 h. Additionally, about 2 g of small intestinal mucosa and 2 g of liver tissue from the left lobe were also collected, placed in 2 mL sterile tubes, and frozen in liquid nitrogen.

### Measurements of hepatic and intestinal histopathology

The fixed liver and intestinal segments were subjected to dehydration using a series of graded ethanol and xylene solutions, followed by embedding in paraffin according to the standard protocol. Subsequently, the embedded tissues were sectioned into 5-mm thin slices utilizing a semi-automatic microtome (Leica Co., Wetzlar, Germany) for hematoxylin and eosin (**H&E**) staining. The morphologies of liver and small intestine were assessed and documented using an Olympus BX51 microscope equipped with a DP70 digital camera (Olympus, Tokyo, Japan).

### Determination of alanine aminotransferase (ALT), inflammatory cytokine and caspase levels

The serum ALT activity was quantified using an assay kit obtained from Nanjing Jiancheng Bioengineering Institute (Nanjing, China). Inflammatory cytokines, including TNF-α, IL-1β, IL-6, and IL-10, in the liver and intestine, as well as IL-18 and NLRP3 in the liver, were analyzed using chicken ELISA kits from Solarbio Science & Technology Co., Ltd. (Beijing, China), according to the manufacturer's guidelines. The liver caspase-1 and caspase-3 activities were detected using ELISA kits (Beyotime Biotechnology, Shanghai, China) as previously described by Liu et al. (2024).

### Determination of intestinal permeability and apoptosis

Serum d-lactate and diamine oxidase (**DAO**) levels were measured using chicken-specific ELISA kits obtained from Meimian Industrial Co., Ltd. (Yancheng, China) (Wang et al., 2022). The TUNEL assay was employed to evaluate the enterocytic apoptosis (Liu et al., 2024), and the stained images were obtained using a laser scanning microscope (LSM700, Carl Zeiss, Oberköchen, Germany). The number of total cell count (blue) and apoptotic cells (green) were calculated with the Image-Pro Plus 6.0 software (Datacell Ltd., Yately, Hampshire, UK).

### Statistical analyses

The individual broiler was considered as the experimental unit for data analyses using one-way ANOVA in SAS 9.4 (Institute Inc., Cary, NC, United States). Mean differences were assessed with Tukey's multiple comparison test, and values were expressed as means ± SE. The differences among group means were analyzed using the least significant difference (**LSD**) method. Significant difference is denoted as *P* < 0.05, and a value of 0.05 ≤ *P* < 0.10 is interpreted as a trend toward significance.

## Results and discussion

**Fig. 1:**
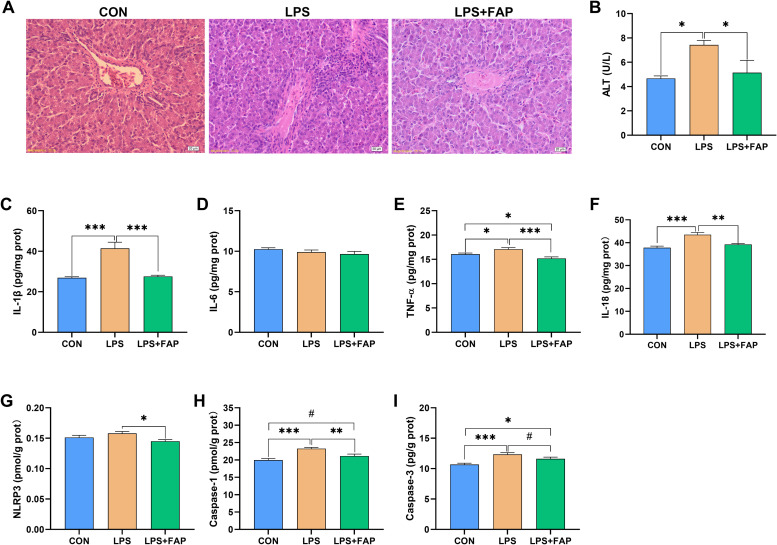
Effects of dietary formic acid polymer (FAP) supplementation on liver morphology, inflammatory cytokines, and caspase activities in broilers challenged with lipopolysaccharide (LPS). (A) Hematoxylin and eosin photomicrographs obtained at 100× magnification; (B) Serum ALT; (C-I) IL-1β, IL-6, TNF-α, IL-18, NLRP3, caspase-1, and caspase-3 in the liver. CON, broilers fed the basal diet and given intraperitoneal administration of saline solution; LPS, broilers fed the basal diet and given intraperitoneal administration of LPS; LPS+FAP, broilers fed the basal diet supplemented with 1,000 mg/kg FAP and given intraperitoneal administration of LPS. *P* < 0.05 was considered statistically significant. * *P* < 0.05, ** *P* < 0.01, *** *P* < 0.001, # 0.05 ≤ *P* < 0.1. *n* = 6.

In the present study, relative to the CON group, the LPS group exhibited increased lymphocyte gathering around the blood vessel, more irregular hepatic cords with some nuclei lysed, and swollen hepatocytes which resulted in narrower hepatic sinusoids (Fig. 1A). Besides, serum ALT activity (Fig. 1B) was significantly elevated in the LPS group compared to the CON group (*P* < 0.05). Serum ALT concentration is considered a sensitive and reliable marker of liver injury (Han et al., 2020). These results indicated that the liver of broiler was damaged by LPS injection. In contrast, dietary FAP supplementation reduced lymphocyte accumulation around the blood vessel, enlarged the hepatic sinusoids, and improved the regularity of the hepatic cords compared with the LPS group. Moreover, FAP supplementation markedly inhibited the LPS-induced increase in serum ALT activity to level observed in the CON group (*P* > 0.05). The above results demonstrated that dietary FAP supplementation could alleviate LPS-induced liver injury of broilers.

Inflammation and cell death are recognized as two major inducers to liver injury (Hsu and Schnabl, 2023; Tao et al., 2023). In this study, LPS challenge significantly increased (*P* < 0.05) liver levels of IL-1β (Fig. 1C), TNF-α (Fig. 1E), and IL-18 (Fig. 1F). Han et al. (2020) also indicated that LPS injection elevated liver pro-inflammatory cytokines concentrations and serum ALT level in broilers. The IL-1β, TNF-α, and IL-18 are three important pro-inflammatory cytokines, and are responsible for a wide spectrum of signaling activities inside cells, resulting in apoptosis, pyroptosis, or necrosis (Kesavardhana et al., 2020). Caspase-1 and caspase-3 have been extensively implicated in the process of cell death, with caspase-1 initiating pyroptosis and caspase-3 driving apoptosis (Kesavardhana et al., 2020). Excessive activation of these caspases can result in tissue damage. Consistently, significantly increased (*P* < 0.05) liver caspase-1 (Fig. 1H) and caspase-3 (Fig. 1I) activities were found in the LPS broilers. However, dietary FAP addition significantly alleviated the increases in liver IL-1β and IL-18 concentrations to levels observed in the CON group (*P* > 0.05), and significantly decreased the levels of TNF-α and NLRP3 (Fig. 1G) in the liver compared with the LPS group (*P* < 0.05). Wang et al. (2022) also found that FAP supplementation decreased the concentrations of IL-1β, TNF-α, IL-18, and NLRP3 in the liver of broilers. The NLRP3 inflammasome is a multimeric cytosolic protein complex that activates caspase-1, which in turn induces the maturation of IL-1β and IL-18, triggering pyroptosis in damaged cells (Kesavardhana et al., 2020). Consistently, LPS+FAP group had significantly lower caspase-1 activity (*P* < 0.05), and tended to decrease the activity of caspase-3 (*P* < 0.10) in the liver compared with the LPS group. Above all, our findings demonstrated that dietary FAP supplementation could suppress cell death by inactivating inflammatory response, thus alleviating LPS-induced liver injury of broilers.

**Fig. 2:**
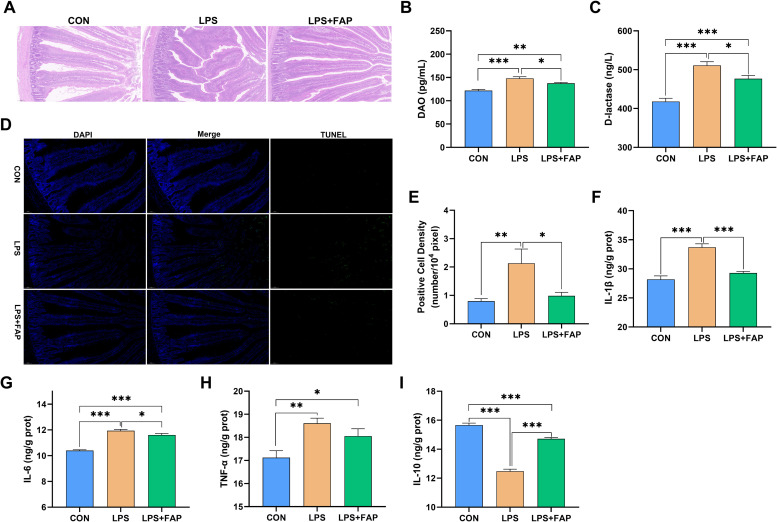
Effects of dietary formic acid polymer (FAP) supplementation on intestinal morphology, permeability, apoptosis, and inflammatory cytokines in broilers challenged with lipopolysaccharide (LPS). (A) Hematoxylin and eosin photomicrographs obtained at 100× magnification; (B, C) DAO and d-lactate in the serum; (D) TUNEL assay of small intestine; (E) Statistical analysis of positive cell density in small intestine; (F-I) Intestinal IL-1β, IL-6, TNF-α, and IL-10. CON, broilers fed the basal diet and given intraperitoneal administration of saline solution; LPS, broilers fed the basal diet and given intraperitoneal administration of LPS; LPS+FAP, broilers fed the basal diet supplemented with 1,000 mg/kg FAP and given intraperitoneal administration of LPS. *P* < 0.05 was considered statistically significant. * *P* < 0.05, ** *P* < 0.01, *** *P* < 0.001. *n* = 6.

The liver and gut engage in extensive communication through the portal vein, biliary tract, and systemic circulation, a bidirectional interaction referred to as the gut-liver axis (Hsu and Schnabl, 2023). Disruption of gut homeostasis is a key contributor to the development of various liver diseases. Tao et al. (2023) showed that repairing intestinal barrier and maintaining gut health could effectively alleviate liver injury in broilers. Intestinal morphological analyses showed that LPS challenge stimulated obvious intestinal villi breakage and shedding, which was evidently abolished by FAP supplementation (Fig. 2A). Serum DAO and d-lactic acid serve as significant indicators of intestinal permeability, and elevated serum levels of these markers suggest increased intestinal permeability and a compromised integrity of intestinal barrier (Liu et al., 2024; Tao et al., 2023). In this study, the serum concentrations of DAO (Fig. 2B) and d-lactate (Fig. 2C) were significantly elevated in the LPS group relative to the CON group (*P* < 0.05), suggesting damaged intestinal mucosal barrier of broiler by LPS challenge. Consistent results were also found in Liu et al. (2024). The broilers in the LPS+FAP group had significantly higher serum DAO and d-lactic acid concentrations than those in the CON group (*P* < 0.05), but significantly lower concentrations than those in the LPS group (*P* < 0.05). It suggested that dietary FAP supplementation alleviated LPS-induced intestinal barrier injury, which might partially contribute to the protective effects of FAP on the liver in LPS-challenged broilers.

Excessive apoptosis of epithelial cells is regarded a significant factor to the damage of intestinal mucosal barrier (Li et al., 2018). Consistently, the TUNEL results (Fig. 2D, E) indicated that the quantity of apoptotic cells in the LPS group was the highest, and the density of positive cells was significantly higher in the LPS groups than in the other two groups (*P* < 0.05), which was in consistent with the results in previous study (Liu et al., 2024). But dietary FAP supplementation significantly inhibited LPS-induced increase in the density of positive cells to level observed in the CON group (*P* > 0.05), demonstrating that dietary FAP addition inhibited LPS-induced intestinal apoptosis in broilers. Inflammatory response is a key factor in LPS-induced apoptosis of intestinal cells (Li et al., 2018). In the present study, LPS injection resulted in significantly higher levels of intestinal IL-1β (Fig. 2F), IL-6 (Fig. 2G), and TNF-α (Fig. 2H) (*P* < 0.05), and significantly lower concentration of intestinal IL-10 (Fig. 2I) (*P* < 0.05) compared to the CON group. Unlike the pro-inflammatory factors IL-1β, IL-6, and TNF-α, IL-10 is an anti-inflammatory cytokine, and plays a central role in infection by regulating the immune response to pathogens, thereby preventing host tissue damage (Kesavardhana et al., 2020). The broilers in the LPS+FAP group also showed significantly higher (*P* < 0.05) IL-6 and TNF-α concentrations and significantly lower (*P* < 0.05) IL-10 content in the small intestine than the broilers in the CON group. However, FAP addition significantly suppressed the alterations in intestinal IL-1β, IL-6, TNF-α, and IL-10 caused by LPS challenge (*P* < 0.05). Therefore, our findings demonstrated that dietary supplementation with FAP could mitigate intestinal apoptosis through inhibiting inflammatory response, which protected the intestinal barrier from LPS-induced damage in broilers.

Collectively, LPS challenge caused inflammatory injury in the liver and small intestine, but dietary 1,000 mg/kg FAP supplementation mitigated LPS-induced damage to the gut-liver axis of broilers through suppressing inflammatory response and cell death. Our findings lay the foundation for future applications of FAP as a feed supplement in preventing liver and intestinal inflammatory diseases in broilers within modern poultry farming.

## Disclosures

The authors declare that they have no known competing financial interests or personal relationships that could have appeared to influence the work reported in this paper.
